# Meibomian Gland Density: An Effective Evaluation Index of Meibomian Gland Dysfunction Based on Deep Learning and Transfer Learning

**DOI:** 10.3390/jcm11092396

**Published:** 2022-04-25

**Authors:** Zuhui Zhang, Xiaolei Lin, Xinxin Yu, Yana Fu, Xiaoyu Chen, Weihua Yang, Qi Dai

**Affiliations:** 1 School of Ophthalmology and Optometry, The Eye Hospital of Wenzhou Medical University, 270 Xueyuanxi Road, Wenzhou 325027, China; zhzhang@eye.ac.cn (Z.Z.); xinxinyu@eye.ac.cn (X.Y.); fuyana@eye.ac.cn (Y.F.); xiaoyuchenny@163.com (X.C.); 2 Department of Ophthalmology and Visual Science, Eye, Ear, Nose, and Throat Hospital, Shanghai Medical College, Fudan University, Shanghai 200126, China; 19111260013@fudan.edu.cn; 3 Affiliated Eye Hospital, Nanjing Medical University, No.138 Hanzhong Road, Nanjing 210029, China; 4 College of Mathematical Medicine, Zhejiang Normal University, Jinhua 321004, China

**Keywords:** meibomian gland dysfunction, meibomian gland density, deep learning, transfer learning, artificial intelligence

## Abstract

We aimed to establish an artificial intelligence (AI) system based on deep learning and transfer learning for meibomian gland (MG) segmentation and evaluate the efficacy of MG density in the diagnosis of MG dysfunction (MGD). First, 85 eyes of 85 subjects were enrolled for AI system-based evaluation effectiveness testing. Then, from 2420 randomly selected subjects, 4006 meibography images (1620 upper eyelids and 2386 lower eyelids) graded by three experts according to the meiboscore were analyzed for MG density using the AI system. The updated AI system achieved 92% accuracy (intersection over union, IoU) and 100% repeatability in MG segmentation after 4 h of training. The processing time for each meibography was 100 ms. We discovered a significant and linear correlation between MG density and ocular surface disease index questionnaire (OSDI), tear break-up time (TBUT), lid margin score, meiboscore, and meibum expressibility score (all *p* < 0.05). The area under the curve (AUC) was 0.900 for MG density in the total eyelids. The sensitivity and specificity were 88% and 81%, respectively, at a cutoff value of 0.275. MG density is an effective index for MGD, particularly supported by the AI system, which could replace the meiboscore, significantly improve the accuracy of meibography analysis, reduce the analysis time and doctors’ workload, and improve the diagnostic efficiency.

## 1. Introduction

Meibomian gland dysfunction (MGD) is a chronic, diffuse abnormality of the meibomian glands (MGs), commonly characterized by terminal duct obstruction and/or qualitative/quantitative changes in glandular secretion and also a major cause of dry eye [1,2]. It can cause tear film instability and ocular surface inflammation, resulting in ocular irritation symptoms, and may even damage the cornea and affect visual function in severe cases. In the absence of a gold-standard diagnostic test, finding effective diagnostic parameters for MGD is imperative. Currently, an intuitive index for assessing MGD is the degree of MG atrophy, which is both common and subjective. In addition, morphological changes in the MGs can also predict the severity of MGD [3,4]. Studies have also confirmed that morphological indices of the MGs, such as their length, width, and tortuosity, are related to their function [5,6]. Ban et al. found that MG morphology in the upper eyelid was significantly correlated with the condition of the tear film or ocular surface epithelium [4]. 

MG atrophy grading has been proven to be an effective diagnostic index for MGD [7,8,9,10]. Based on the findings of these studies, further studies used ImageJ and other software to manually label the MG for a quantitative analysis. However, manual labeling of MGs is subject to insurmountable subjective errors and is time-consuming, resulting in low efficiency. 

In subsequent studies, image-processing algorithms have become popular research tools for MG image analysis. Some analytical methods showed superiority in MG morphological analysis. Arita et al. reported an image processing system that could analyze the MG morphology and obtain relatively accurate results [11,12]. Llorens-Quintana et al. reported a new methodology for analyzing, in an automated and objective fashion, infrared images of the MG [13]. Ciężar et al. reported that global 2D Fourier transform analysis of infra-red MG images provides values of two new parameters: mean gland frequency and anisotropy in gland periodicity. Their values correlate with MGD [14]. Yeh et al. reported a nonparametric instance discrimination approach that automatically analyses MG atrophy severity from meibography without prior image annotations and categorizes the MG characteristics through hierarchical clustering [15]. However, traditional image algorithms still have some limitations, such as unstable region detection and weak characterization of the extracted features. In addition, the overall evaluation index is based on the dropout grade classification of MGs, and it is impossible to extract and analyze each gland separately [16]. 

Previous studies on artificial intelligence (AI), such as convolutional neural networks, have proven effective in the automatic evaluation of meiboscore [17,18,19]. However, these studies focused on MG dropout grade classification and did not segment each MG. Consequently, they could not be further analyzed. 

The purpose of this study was to develop an AI-based evaluation system for MG morphology based on deep learning and transfer learning for segmenting each MG and evaluating MG morphological indices accurately. Furthermore, the study also aimed to make it possible to diagnose MGD using MG density, an index that requires many annotations and calculations.

## 2. Materials and Methods

### 2.1. Patients and Materials

The subjects used in the AI model training were the same as those in our previous report [20], and a total of 60 randomly selected subjects were recruited. Sixty original annotated meibography images of the upper eyelids were used in this study. Of these, 40 were used as the original training images. A total of 245,760 images were generated from these 40 images as a training set using image enhancement software. Another 20 annotated meibography images were used as validation sets. Subsequently, we adjusted the parameters and trained the AI to apply it to the lower eyelid. Sixty original annotated meibography images of the lower eyelids were used for the validation.

First, 85 eyes from 85 subjects (age, 8–83 years) were enrolled for the AI system analysis and evaluation of the efficacy of MG density for MGD diagnosis. Only one eye of each subject was randomly selected and included for the comprehensive dry eye and MG examination. The exclusion criteria were as follows: (1) history of ocular trauma or surgery; (2) systemic drugs or eye drops affecting MG function or tear film used in the last 2 weeks; (3) contact lenses worn in the last 2 weeks; and (4) ocular or systemic diseases known to affect tear film or MG function. A total of 53 subjects with obstructive MGD (20 males and 33 females; median age, 35.00 (30.00–50.00) years) were included in the MGD group, and 32 healthy subjects (13 males and 19 females; median age, 25.00 (16.25–32.75) years) in the control group. 

All 53 subjects with obstructive MGD were diagnosed by two experienced ophthalmologists when any two of the three scores were abnormal: (I) ocular symptom score ≥ 3; (II) lid margin abnormality score ≥ 2; and/or (III) meiboscore ≥ 3 [21]. Subjects diagnosed with obstructive MGD by both ophthalmologists were included in this study. If the ophthalmologists provided different diagnoses, the subjects were excluded from the study. 

A total of 4006 meibography images (including 1620 upper eyelids and 2386 lower eyelids) from 2420 randomly selected subjects (age ≥18 years) were used for MG density analysis using the AI system. All 4006 meibography images were graded according to the meiboscore (range, 0–3) by three experienced ophthalmologists, and their majority opinion was obtained. A qualified meibography image needed to meet two requirements: (1) the tarsal plates must be entirely exposed, and (2) the meibography image must be focused correctly and clearly. Unqualified meibography images would interfere with the meiboscore and MG density results. The correlation between MG density obtained by the AI system and meiboscore from ophthalmologists was analyzed.

All subjects were from the Eye Hospital, Wenzhou Medical University. The study was conducted in accordance with the Declaration of Helsinki and was approved by the Research Ethics Committee of the Eye Hospital, Wenzhou Medical University (approval number: 2020-209-K-191). This study is registered on http://www.chictr.org.cn (ChiCTR2100052575, 31 October 2021). Informed consent to publish was obtained from all participants before their inclusion in the study.

### 2.2. Methods

#### 2.2.1. Data Collection and Processing of Samples

Samples were collected, optimized, and processed using the previously reported method [20]. First, images of both the upper and lower MGs were captured using the Oculus Keratograph 5M (K5M; Oculus, Wetzlar, Germany). Second, these images were optimized, converted to grayscale, and then standardized and normalized.

#### 2.2.2. Network Structure and AI Training

The tarsus segmentation model was based on Mask R-CNN [22]. Based on the pre-trained Mask R-CNN model (https://github.com/matterport/Mask_RCNN, 20 March 2018), we used 100 annotated images of upper and lower tarsus for fine-tuning and obtained fine-tuned model parameters after iterating 200 epochs. Another 20 sample images were used to test the fine-tuned model. Finally, we used the fine-tuned Mask R-CNN model to segment the tarsus. 

**Figure 1:**
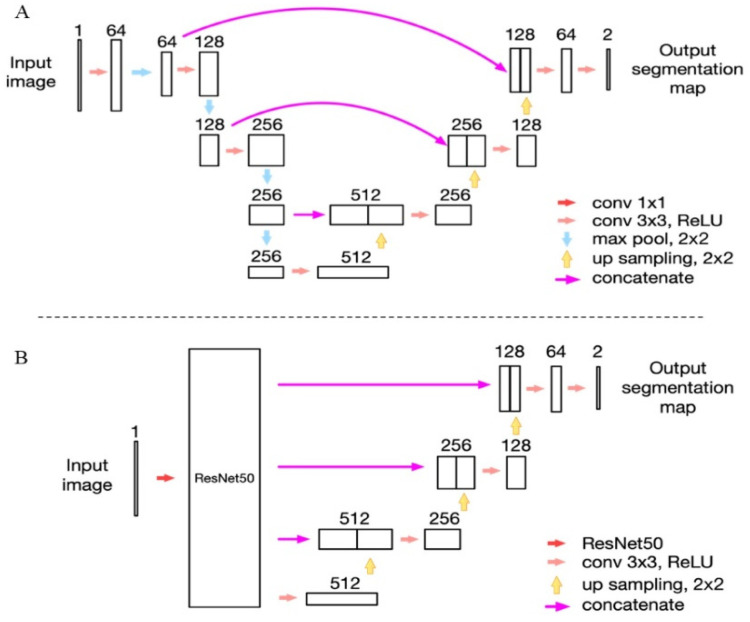
**Network Structure.** (**A**) The network structure of the modified U-net model as we reported previously; (**B**) the network structure of the ResNet50_U-net model in this study.

Transfer learning was used to apply the pretrained model and parameters on ImageNet [23] to our previously reported deep learning model (Figure 1A). The residual neural network (ResNet) exhibits excellent performance in image classification and target detection [24]. The 50-layer ResNet (ResNet50) was replaced with the max-pooling layers of the previous U-net model; however, the upsampling layer remained the same (Figure 1B). We call this the ResNet50_U-net. 

Forty annotated meibography images of the upper eyelids were included as the basis for the training set. In each iteration of training, four images of these 40 original meibography images were randomly selected. The data enhancement model (https://github.com/aleju/imgaug#citation, 6 February 2020, Figure 2) was used to enhance the input of four images with random use of algorithms and parameters, with four new images generated. The final version of the model was iterated a total of 61,440 times in all training and generated 245,760 new images as the training set. The amount of data can preliminarily meet the needs of training a deep convolutional neural network. 

**Figure 3:**
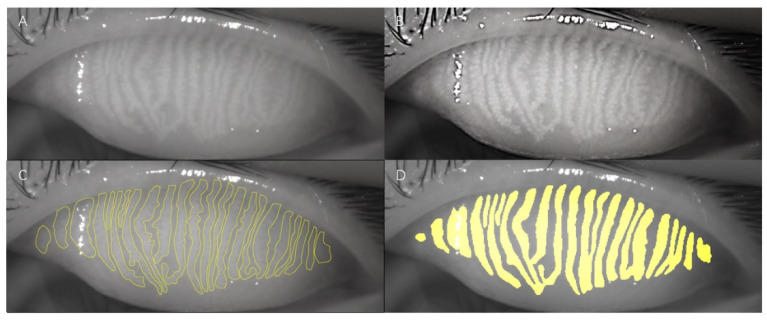
**The samples of image processing, annotation and segmentation.** (**A**) The original meibography. (**B**) The preprocessed image. (**C**) The manually annotated MGs in yellow outline. (**D**) The segmented MGs by AI system are shown in yellow.

**Figure 4:**
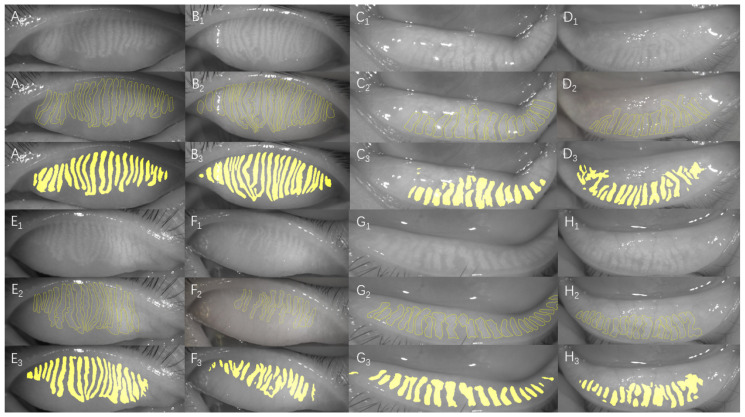
**The comparisons of manual annotation and AI automatic segmentation.** (**A_1_**,**B_1_**,**E_1_**,**F_1_**) The original meibography of the upper eyelid. (**A_2_**,**B_2_**,**E_2_**,**F_2_**) The manual annotation of the upper eyelid (yellow outline). (**A_3_**,**B_3_**,**E_3_**,**F_3_**) The AI segmentation MGs of the upper eyelid (yellow part). (**C_1_**,**D_1_**,**G_1_**,**H_1_**) the Original meibography of the lower eyelid. (**C_2_**,**D_2_**,**G_2_**,**H_2_**) The manual annotation of the lower eyelid (yellow outline). (**C_3_**,**D_3_**,**G_3_**,**H_3_**) The AI segmentation MGs of the lower eyelid (yellow part).

The original meibography (Figure 3A) was preprocessed to show the glands more clearly (Figure 3B). Compared to the manually annotated result (Figure 3C), the AI system exhibited superior recognition ability (Figure 3D). Figure 4 shows a sample of the original meibography, manual annotation, and AI segmentation of the MGs.

**Figure 5:**
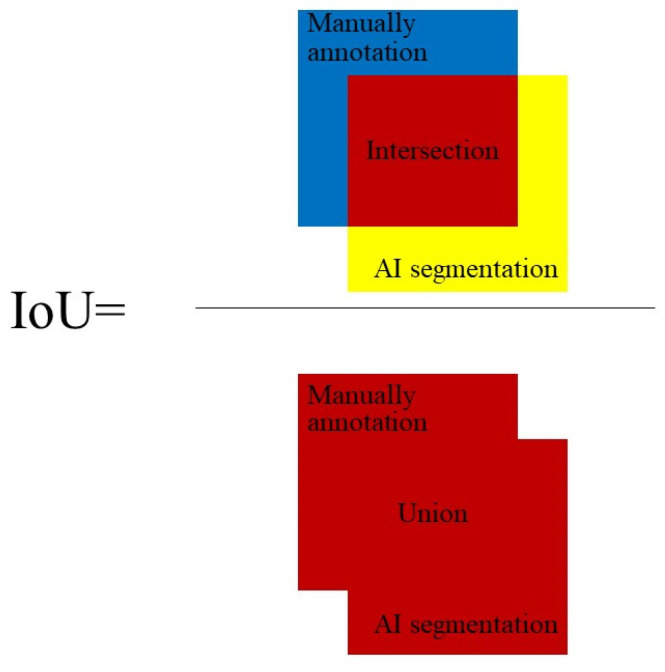
The intersection (green part) of the ground truth (manual annotation, blue part) and the AI result (AI segmentation, yellow part) divided by their union (red part) is IoU.

We used another 20 annotated original upper eyelid meibography images apart from the training set as the validation set. We used the intersection of unions (IoU) to evaluate the accuracy of the MG recognition model (Figure 5). It can be simply understood as the ratio of the intersection of the ground truth (manual annotation) and AI result (AI segmentation) to their union.

#### 2.2.3. Clinical Parameters

The clinical assessments were performed sequentially as follows [20]. All subjects completed the Ocular Surface Disease Index (OSDI) questionnaire and were asked whether they had any of the 14 MGD-related ocular symptoms (symptom score) [25]. Images of both the upper and lower MGs were captured using the Keratograph 5M. The central tear meniscus height (TMH) of the lower eyelid was measured 5 s after blinking using the Keratograph 5M. Tear break-up time (TBUT) was measured and corneal fluorescein staining (CFS) was performed after the instillation of fluorescein. TBUT was measured three times, and the mean value was recorded. CFS was graded according to the Baylor grading scheme from 0 to 4 [26]. Four lid margin abnormalities (irregular lid margin, vascular engorgement, plugged meibomian gland orifices, and anterior or posterior replacement of the mucocutaneous junction) were scored from 0 to 4, according to the number of these abnormalities present in each eye [21]. The MG expressibility scores ranged from 0 to 45 by assessing the meibum quality and quantity of the 15 glands on each lower eyelid [27]. 

#### 2.2.4. MG Indices

To assess the degree of MG dropout, we used the method described by Arita et al. to calculate the meiboscore: 0, no loss of MGs; 1, the lost area was less than one-third of the total area of the MGs; 2, the lost area was between one-third and two-thirds of the total area of the MGs; and 3, the lost area was over two-thirds of the total area of the MGs [9]. The total meiboscore of the upper and lower eyelids ranged from 0 to 6. 

MG density was automatically calculated by the AI system using the following formula [28]: the sum of the area of MGs divided by the total area of the tarsus in pixels. $\sum_{i=1}^{n}S_{MG_{i}}$ = the sum of pixels of all MGs, St = the total pixels of the tarsus.

$$MG\;density=\frac{\sum_{i=1}^{n}S_{MG_{i}}}{St}$$

#### 2.2.5. Statistical Analysis

The normality of data distributions was analyzed using the Kolmogorov–Smirnov test, and the abnormal data distributions were analyzed using the non-parametric statistical analyses. Values are expressed as the mean ± standard deviation (SD) or (range) or median (interquartile range [IQR]). Either the independent samples t-test or the Mann–Whitney U-test was used to compare differences between MGD subjects and normal control subjects. The generalized estimating equation was used to adjust the age difference. Kruskal–Wallis H-test was used to compare the MG density and the severity score of the meiboscore scale. The correlations between various MG morphological parameters and MG function parameters (i.e., OSDI, TBUT, CFS, lid margin score, meiboscore, and meibum expressibility score) were determined using Pearson’s or Spearman’s correlation analysis. The χ2 test was used to compare the sex ratios between the two groups. Receiver operating characteristic (ROC) curve analysis was used to determine the predictive value of MG density for the diagnosis of MGD. A two-sided *p* < 0.05 was considered statistically significant. All statistical analyses were performed using SPSS Statistics 23.0 (IBM, Armonk, NY, USA).

## 3. Results

### 3.1. AI Training and Testing

The AI system of Mask R-CNN achieved 93% accuracy (IoU) and 100% repeatability for tarsus segmentation. The AI system of ResNet50_U-net training lasted for 4 h, a significant reduction from the duration of our previous U-Net model training (15 h). Additionally, the ResNet50_U-net model achieved 92% accuracy (IoU) and 100% repeatability for MG segmentation. Subsequently, we adjusted the parameters and trained the AI to automatically segment the lower MGs and achieved the same level of IoU. The processing time of each meibography was 100 ms with a GTX 1070 8G GPU.

### 3.2. Characteristics

A total of 85 eyes from 85 randomly selected subjects were enrolled for AI system effectiveness testing. These included 53 subjects with obstructive MGD (20 males and 33 females, median age, 35.00 (30.00–50.00) years) and 32 normal volunteers (13 males and 19 females, median age, 25.00 (16.25–32.75) years). Because the age difference between patients with MGD and the normal control group was significant, the generalized estimating equation was used to adjust for age. No significant difference in sex was observed between patients with MGD and normal controls. The baseline characteristics of the 85 subjects are summarized in Table 1. 

### 3.3. MG Density and Functions

The MG density in the upper eyelid was significantly correlated with OSDI (r = −0.320, *p* = 0.003), TBUT (r = 0.484, *p* < 0.001), lid margin score (r = −0.350, *p* = 0.001), meiboscore (r = −0.749, *p* < 0.001), and meibum expressibility score (r = 0.425, *p* < 0.001). The MG density in the lower eyelid was significantly correlated with OSDI (r = −0.420, *p* < 0.001), TBUT (r =0.598, *p* < 0.001), lid margin score (r = −0.396, *p* < 0.001), meiboscore (r = −0.720, *p* < 0.001), and meibum expressibility score (r = 0.438, *p* < 0.001). The MG density in the total eyelid was significantly correlated with OSDI (r = −0.404, *p* < 0.001), TBUT (r = 0.601, *p* < 0.001), lid margin score (r = −0.416, *p* < 0.001), meiboscore (r = −0.805, *p* < 0.001), and meibum expressibility score (r = 0.480, *p* < 0.001). However, there were no significant correlations between MG density and CFS or TMH in upper eyelid, lower eyelid and total eyelid (all *p* > 0.05). These results are shown in Table 2.

### 3.4. MG Density with Meiboscore

**Figure 6:**
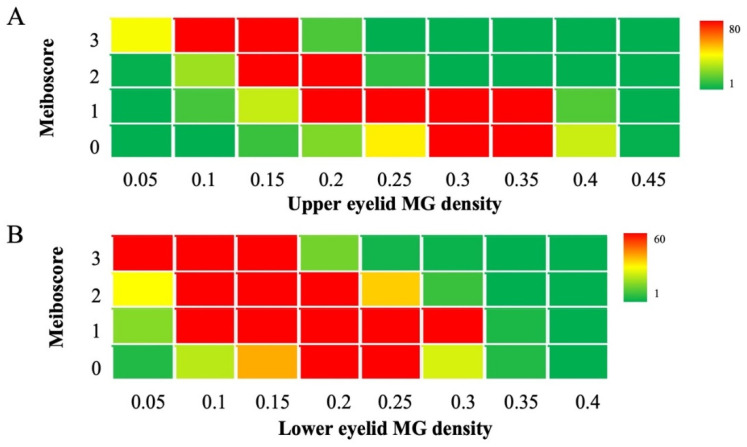
Corresponding relationship between MG density and meiboscore. (**A**) The corresponding relationship between the upper eyelid MG density and meiboscore. (**B**) The corresponding relationship between lower eyelid MG density and meiboscore. The “hot” red areas represent data-intensive areas. The maximum number was 80 and 60 meibography images on the upper eyelid and lower eyelid, respectively. The “cold” green areas are the opposite. The minimum value is 1 meibography image.

After analyzing 4006 random meibography images using the AI system, it was observed that the MG density in the upper eyelid was significantly negatively correlated with the meiboscore (r = −0.707, *p* < 0.001), as was that in the lower eyelid (r = −0.472, *p* < 0.001). The corresponding relationship between the MG density and meiboscore is shown in Figure 6.

### 3.5. MG Density to Meiboscore

**Figure 7:**
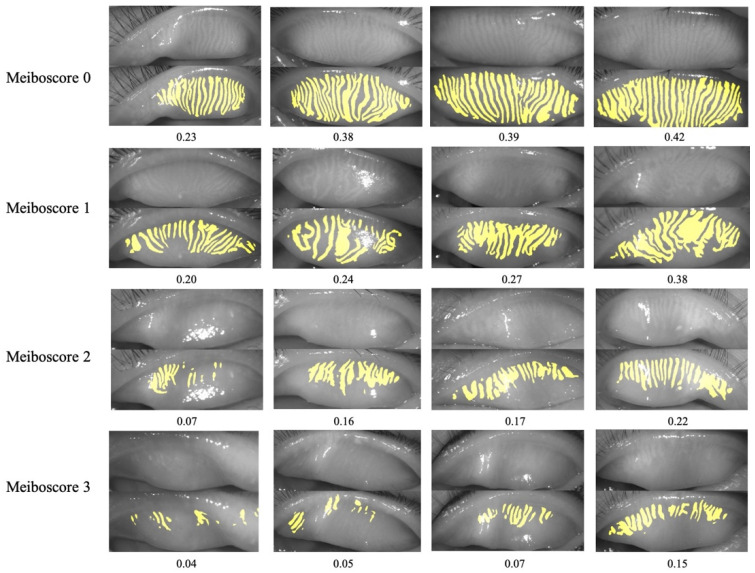
Meibography images with MG densities and meiboscores. Rows 1 to 4 refer to meibography images with meiboscore 0 to 3, respectively. MG density was calculated from the meibography images by our AI system.

We compared the correspondence between the MG density and meiboscore, as shown in Table 3. The MG density distribution in the upper eyelid on each meiboscore scale was not the same, and the difference was significant (H = 882.932, *p* < 0.001). The MG density distribution in the lower eyelid on each meiboscore scale was not the same, and the difference was significant (H = 596.815, *p* < 0.001). Figure 7 depicts meibography images with varying MG densities and corresponding meiboscores to help readers gain insight into the relationship between MG density and meiboscore. 

### 3.6. Sensitivity and Specificity of MG Density

**Figure 8:**
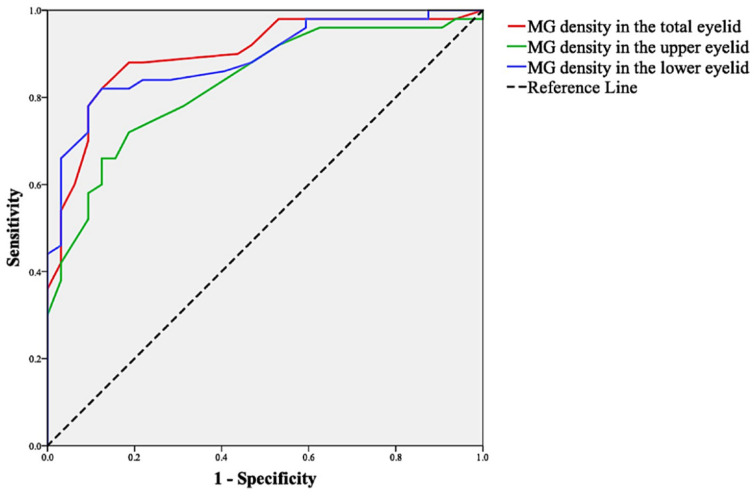
ROC curve analysis of MG density for the diagnosis of MGD.

Figure 8 shows the results of the ROC curve analyses, which indicated the sensitivity and specificity of MG density for the diagnosis of MGD. The area under the curve (AUC) was 0.836 for MG density in the upper eyelid. The sensitivity and specificity were 73% and 81%, respectively, at a cut-off value of 0.265. The AUC was 0.888 for MG density in the lower eyelid. The sensitivity and specificity were 82% and 88%, respectively, at a cut-off value of 0.255. The AUC was 0.900 for MG density in the total eyelids. The sensitivity and specificity were 88% and 81%, respectively, at a cut-off value of 0.275.

## 4. Discussion

Diagnosis of MGD is difficult because most of the diagnostic criteria are subjective and are usually based on a combination of a high meiboscore, dry eye symptoms, and lid margin abnormalities [29]. A comprehensive analysis of MG morphology is the key for determining the severity of MGD. Currently, the most widely used MG morphology criterion is a qualitative MG dropout grading index similar to the meiboscore, and its effectiveness has been proven by a large number of studies. However, the meiboscore and other qualitative grading indices also have the limitations of strong subjectivity and poor repeatability, especially regarding the results adjacent to the grading transition zone. For example, when the MG dropout ratio is 1/3 or 2/3, the meiboscore becomes unstable. This study proposes a novel MG dropout index, the MG density. It is a linear quantitative index that extracts the image of each MG gland and calculates the ratio of the precise gland area relative to the tarsus area. This novel index greatly improves the accuracy compared with the traditional MG atrophy grade method, but it also shows instability and inaccuracy owing to anthropogenic annotation errors, limiting its effectiveness when using manual calculations. This MG density index requires many calculations, which limits its clinical application. AI has a quick mathematical calculation ability and high reliability, which is suitable for calculating MG density. There was no need to control between-group variance and repeatability, such as within-subject SD (SW), within-subject coefficient of variation (CVw), and intraclass correlation coefficient (ICC), as in our previous study [20,28]. In this study, the AI system achieved a 92% IoU and 100% repeatability. 

The AI model used in this study is the latest iteration of the CNN model used in our previous study [20]. To further improve the recognition accuracy of AI systems, a large training dataset is required. To overcome the dilemma of fewer MG images, we selected a data enhancement model to manipulate MG images and a combination of deep and transfer learning for AI model building. Transfer learning techniques attempt to transfer knowledge from previous tasks to a target task when the latter has less high-quality training data. This can be accomplished using a network that has already been pretrained on millions of general-purpose images (ImageNet [30]) without any additional retraining needed for the deep convolutional neural networks on our specific dataset. Using transfer learning, we were able to use a pretrained neural network in our image recognition network, which greatly reduced the dependence on training data and improved the training speed (from 15 h for the U-Net model to 4 h for the ResNet50_U-net model) and accuracy. Transfer learning has been used to study ophthalmic diseases, such as age-related macular degeneration [31] and glaucoma [32]. Although a small number of subjects were used to train AI in this study, the detection accuracy was very high owing to the combination of deep learning and transfer learning. 

After comparing the relationship between the MG morphological indices extracted by the AI system and clinical parameters, as previously reported [3,33,34], the AI system in this study revealed that MG dropout was significantly correlated with MGD symptoms, tear film stability, lid margin abnormality, and meibum expressibility. One step further than previous research [7,9,18,19,35,36], our study used MG density instead of meiboscore to evaluate the degree of MG dropout successfully. ROC curve analysis revealed that MG density showed high diagnostic efficiency for MGD. MG density in the total eyelids showed good efficiency, sensitivity, and specificity for the diagnosis of MGD, with a sensitivity and specificity of 88% and 81%, respectively, at a cut-off value of 0.275.

Furthermore, regarding MG atrophy evaluation, a quantitative index based on the continuous numerical result of MG density is a better criterion than a qualitative index based on the MG dropout grade of the meiboscore. It is difficult to provide precise meiboscores when MG atrophy is near the grading transition limits (0%, 33%, and 66%), whereas MG density can be used in such situations. MG density can be used to effectively assess the atrophy condition of the MG in each grading transition area. Simultaneously, we also proposed the corresponding and conversion relation between the MG density and meiboscore by analyzing 4006 meibography images. There was a significant linear correlation between the MG density and meiboscore, especially in the upper eyelid. The MG density of the lower eyelid was slightly less correlated with the meiboscore, which may be related to the fact that the lower palpebral conjunctiva was mistakenly identified as the tarsus by the AI system because of the excessive turnover of the lower eyelid. In the future, based on AI assistance, the quantitative index of MG density can be used to replace the qualitative index of MG dropout, such as the meiboscore.

This study has some limitations. The sample size for AI training was small. Even though we used imgaug, a data enhancement library, which could partially obtain a large amount of information from the original meibography used for AI training and greatly reduce the workload of annotation, it still could not change some basic information of the meibography, such as the number of glands. Therefore, it could not completely replace the newly annotated images. In addition, the sample size for evaluating the diagnostic efficacy of MG density was small. In future studies, the author’s team will recruit more subjects for AI system training and testing. 

## 5. Conclusions

MG density is an accurate and effective evaluation index that can completely replace the meiboscore for the quantitative diagnosis of MG dropout. We propose MG density as a novel quantitative index for AI-based diagnosis of MGD. Simultaneously, the AI system can reduce the subjective bias of the observer and doctors’ workload, improve efficiency, and assist nonprofessional doctors with MGD diagnosis.

## Figures and Tables

**Figure 2:**
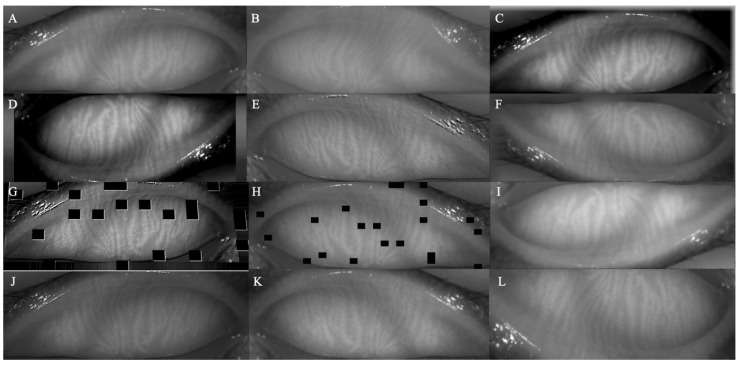
**Theimage enhancement modelexample.** (**A**) Original training image. (**B**–**L**) Output images with 11 enhancement methods.

**Table 1:** Clinical parameters of the 85 subjects.

Parameter	Normal (n = 32)	MGD (n = 53)	*p*	*p* *
Age (years), Median (IQR)	25.00 (16.25–32.75)	35.00 (30.00–50.00)	<0.001	-
Sex (n, male/female)	13/19	20/33	0.794	-
OSDI (0–100), Median (IQR)	4.47 (0.30–12.35)	25.00 (13.24–37.80)	<0.001	<0.001
Symptom score (0–14), Median (IQR)	2.00 (0–4.00)	7.00 (5.00–8.00)	<0.001	<0.001
TBUT (s), Median (IQR)	5.00 (5.00–7.75)	2.50 (1.33–3.67)	<0.001	<0.001
CFS (0–20), Median (IQR)	0 (0–0)	0 (0–0)	0.058	0.021
TMH (mm), Median (IQR)	0.19 (0.16–0.23)	0.20 (0.17–0.24)	0.461	0.871
Lid margin score (0–4), Median (IQR)	0 (0–1.00)	2.00 (1.00–2.00)	<0.001	<0.001
Meiboscore (0–6), Median (IQR)	2.00 (1.00–2.00)	3.00 (2.00–4.50)	<0.001	<0.001
Meibum expressibility score (0–45), Median (IQR)	38.50 (30.00–45.00)	18.00 (5.50–34.50)	<0.001	<0.001

MGD = meibomian gland dysfunction; IQR = interquartile range; OSDI = Ocular Surface Disease Index; TBUT = tear break-up time; CFS = corneal fluorescein staining; TMH = tear meniscus height; Values are expressed as the median (IQR). Mann–Whitney U-test was used to compare differences between MGD subjects and normal control subjects. * *p* values adjusted for age by generalized estimating equation.

**Table 2:** Correlations of MG density with tear film functions and MG status in 85 subjects.

		OSDI	TBUT	CFS	TMH	Lid Margin Score	Meiboscore	Meibum Expressibility Score
MG density	Upper eyelid	−0.320 †	0.484 ‡	−0.162	−0.059	−0.350 †	−0.749 ‡	0.425 ‡
	Lower eyelid	−0.420 ‡	0.598 ‡	−0.177	−0.058	−0.396 ‡	−0.720 ‡	0.438 ‡
	Total eyelid	−0.404 ‡	0.601 ‡	−0.166	−0.070	−0.416 ‡	−0.805 ‡	0.480 ‡

MG = meibomian gland; OSDI = Ocular Surface Disease Index; TBUT = tear break-up time; CFS = corneal fluorescein staining; TMH = tear meniscus height; Spearman’s rank correlation coefficient test. † *p* < 0.005. ‡ *p* < 0.001.

**Table 3:** Comparison table of MG density and meiboscore.

	MG Density					
	Upper Eyelid (1620)			Lower Eyelid (2386)		
	Median (IQR)	H-Value	*p*	Median (IQR)	H-Value	*p*
Meiboscore 0	0.30 (0.25–0.33)	882.932	<0.001	0.19 (0.14–0.23)	596.815	<0.001
Meiboscore 1	0.25 (0.21–0.29)			0.17 (0.13–0.21)		
Meiboscore 2	0.15 (0.12–0.18)			0.13 (0.10–0.17)		
Meiboscore 3	0.10 (0.06–0.12)			0.07 (0.04–0.11)		

MG = meibomian gland; IQR = interquartile range; *p*: compare distributions across groups; H-value: test statistic.

## Data Availability

The datasets generated during and/or analyzed during the current study are available from the corresponding author on reasonable request.
